# Unveiling the Impact of the Genomic Architecture on the Evolution of Vertebrate microRNAs

**DOI:** 10.3389/fgene.2017.00034

**Published:** 2017-03-21

**Authors:** Gustavo S. França, Ludwig C. Hinske, Pedro A. F. Galante, Maria D. Vibranovski

**Affiliations:** ^1^Departamento de Genética e Biologia Evolutiva, Universidade de São PauloSão Paulo, Brazil; ^2^Department of Anesthesiology, Clinic of the University of Munich, Ludwig Maximilian University of MunichMunich, Germany; ^3^Centro de Oncologia Molecular, Hospital Sírio-LibanêsSão Paulo, Brazil

**Keywords:** intragenic, intergenic region, new and old miRNAs, host gene, target interactions, expression breadth

## Abstract

Eukaryotic genomes frequently exhibit interdependency between transcriptional units, as evidenced by regions of high gene density. It is well recognized that vertebrate microRNAs (miRNAs) are usually embedded in those regions. Recent work has shown that the genomic context is of utmost importance to determine miRNA expression in time and space, thus affecting their evolutionary fates over long and short terms. Consequently, understanding the inter- and intraspecific changes on miRNA genomic architecture may bring novel insights on the basic cellular processes regulated by miRNAs, as well as phenotypic evolution and disease-related mechanisms.

## Introduction

Recent genome-wide projects have revealed an outstanding transcriptome diversity, especially of non-coding RNAs (ncRNAs), as well as a wealth of regulatory mechanisms and gene product interactions that compound the molecular basis of phenotypes (Carninci et al., 2005; Mele et al., 2015). A notable feature that soon became clear is the interleaved nature of eukaryotic genomes, despite their typical large sizes. This means that a particular genomic region can be suited for different purposes, with an extensive overlap of transcriptional units either in sense or antisense DNA strands (Kapranov et al., 2007).

The interleaved model opens up numerous possibilities for regulatory mechanisms. For instance, products of antisense transcription, which is believed to occur in more than 30% of gene loci in humans (Galante et al., 2007), can regulate gene activity through many different ways (reviewed in Pelechano and Steinmetz, 2013). In the interleaved genome, transcription units may show high interdependency, whereby neighboring or overlapping genes can be co-regulated by shared regulatory elements; yet, structural changes in the chromatin environment can also influence their expression coordinately (Mellor et al., 2016). Complex transcriptional networks thus emerge from a modular architecture that can either be shaped by evolutionary advantages and constraints (Mercer and Mattick, 2013), but also as a result of neutral processes (Graur et al., 2015). Such interleaved architecture is particularly striking in regard to microRNAs (miRNAs). Ever since the first large-scale studies on their genomic organization (Rodriguez et al., 2004), it is commonly observed that these small non-coding RNAs overlap to protein-coding genes, with vertebrate miRNAs mapping to intronic regions more than expected by chance (Baskerville and Bartel, 2005; Hinske et al., 2010, 2014; Campo-Paysaa et al., 2011; Meunier et al., 2013). As they comprise an essential class of gene expression regulators in basic biological processes and diseases, genomic context analyses are pivotal to uncover unique aspects of miRNA biology. Here, we discuss recent advances in this topic focusing on the importance of the genomic context to miRNA expression and their target interactions. In this framework, we highlight the evolutionary consequences for the fixation of newly emerged miRNAs and functional properties arising from miRNA–genomic context relationships over long-and short-evolutionary terms.

## The Impact of the Genomic Context on miRNA Expression and Function

As any other gene, the evolutionary processes that gives rise to new miRNAs – mainly by duplication or *de novo* origin (Berezikov, 2011; Meunier et al., 2013) – takes place on certain regions of the genome that may overlap or not to preexisting gene loci. In a recent study, Meunier et al. (2013) showed that all vertebrate species analyzed (Chicken, Platypus, Opossum, Mouse, Macaque, and Human) have a significant excess of intragenic miRNAs, with on average 54% of them overlapping to introns. Curiously, the proportions of intronic miRNAs are even higher for those of recent origin, suggesting that introns are hotspots for new miRNA origination. Moreover, the transcriptional orientation of intragenic miRNAs is highly biased (∼80%) toward the same strand orientation of their host genes (Rodriguez et al., 2004; Campo-Paysaa et al., 2011; Meunier et al., 2013; Hinske et al., 2014).

Given the large size of vertebrate genomes, why do miRNAs apparently have such preference to emerge in intragenic regions? Which evidences support the role of natural selection shaping this pattern, and what advantages miRNAs might take from such genomic organization? To address these questions, França et al. (2016) investigated the patterns of emergence and expression of human miRNAs along the vertebrate evolution considering the evolutionary origin of their host genes, i.e., whether miRNAs are intergenic, mapped to old protein-coding genes (originated before fish and tetrapods divergence), or to young protein-coding genes (originated after the divergence). Similar to previous studies (Iwama et al., 2013), it was shown that most human miRNAs (∼70%) have a relatively recent origin, emerging in the primate order. Though an interesting pattern was revealed, the majority of those young miRNAs are intragenic and preferentially embedded within old host genes, even when controlled by host gene length (including intronic region) and expression level. Expression breadth analyses showed that young miRNAs hosted by old genes were more broadly expressed (expression in more tissues) than their intergenic counterparts. On the other hand, miRNAs hosted by young genes showed a bias to tissue-specific expression when compared to the intergenic ones or those within old genes. The same conclusions held when a very stringent miRNA annotation provided by Fromm et al. (2015) was considered, since several miRBase entries do not represent bonafide miRNAs (Chiang et al., 2010; Taylor et al., 2014; Fromm et al., 2015). It is well established that expression breadth is negatively correlated with evolutionary rates (Wolf et al., 2009; Park and Choi, 2010), meaning that overall conserved genes are highly and broadly expressed, whereas less conserved genes tend to have low and narrow expression. What turns out is that the expression of intragenic miRNAs is tightly coupled to their genomic environment, especially in regard to the evolutionary ages of their host genes. In a mechanistic way, this is clearly connected with the co-expression of miRNA–host gene pairs by shared regulatory elements, a very well-documented event (Baskerville and Bartel, 2005; Ozsolak et al., 2008; Marsico et al., 2013). Hence, the maintenance of miRNAs embedded in genic regions may be indicative of some evolutionary constraint, since young and older intragenic miRNAs are biased toward host gene sense orientation, as well as preferential emergence within old host genes. In addition, same age miRNAs show differential expression breadth depending on their genomic context, a pattern that is maintained not only during recent (e.g., primates) but also over longer periods. Such pattern is observed for miRNAs originated in amniotes (e.g., chicken) or in placental mammals (e.g., mouse) presenting higher or lower expression breadth depending on the age of their host genes (França et al., 2016).

In particular for young intragenic miRNAs, being hosted by old genes could be beneficial at least during an initial adaptive phase, because of the expression broadness achieved through a presumably favorable transcriptional environment. Instead of readily relying on the settlement of their own regulatory apparatus, young miRNAs would initially been benefited by their hosts’ regulatory elements, albeit they may acquire independent regulation afterward (França et al., 2016). Supporting this notion, it has been suggested that young and middle-aged intragenic miRNAs are more likely to be regulated by shared promoters, whereas old miRNAs are frequently regulated by their independent intronic promoters (Marsico et al., 2013). In addition, as old host genes provide higher expression breadth for those young miRNAs, it would, in principle, increase the opportunities for new target interactions in different tissues. From such perspective, the host transcriptional environment could facilitate the initial expression of young miRNAs and thereafter contribute to the process of miRNA functionalization.

The location of a gene in the genome is clearly related to its expression, as revealed by transgene insertion experiments (Mlynárová et al., 2002) and global expression analyses of gene neighborhoods (Caron et al., 2001; Purmann et al., 2007; Michalak, 2008). Nevertheless, some of the observed expression changes in gene vicinity may not be subjected to selection, but rather it would be a consequence of expression changes in a close gene under strong selection. Recently, Ghanbarian and Hurst (2015) demonstrated that expression changes in humans, relative to the human–chimp common ancestor, coordinately drive changes in expression of the neighbors of a focal gene, and that this effect is stronger as the distance between genes are shorter (<100kbp). Therefore, the genomic context still may yield important effects on the expression, and perhaps the fixation of novel miRNAs that are not under direct selection.

## Evolutionary Conservation and Novelties From miRNAs’ Genomic Context

The phylogenetic distribution of miRNAs in vertebrates is distinguished by the presence of deeply conserved and abundant clade or species-specific repertoires (Berezikov et al., 2006; Wheeler et al., 2009; Meunier et al., 2013; Fromm et al., 2015). Although the evolution of miRNA sequences have been investigated (Lyu et al., 2014; Ninova et al., 2014), the conserved patterns and evolutionary innovations that arose due to interspecific differences in the genomic context are largely underexplored. One of the few studies to address this issue compared the genomic location and expression of ∼100 miRNAs during developmental stages of medaka fish, zebrafish, chicken, and mouse (Ason et al., 2006). It was demonstrated that spatial expression differences can be related to changes either in the miRNA location and copy number variation rather than to sequence divergence (Ason et al., 2006). Actually, the miRNA genomic location is thought to influence their expression divergence, as old- and middle-aged intragenic miRNAs tend to be more similarly expressed among species than intergenic ones (França et al., 2016).

**FIGURE 1:**
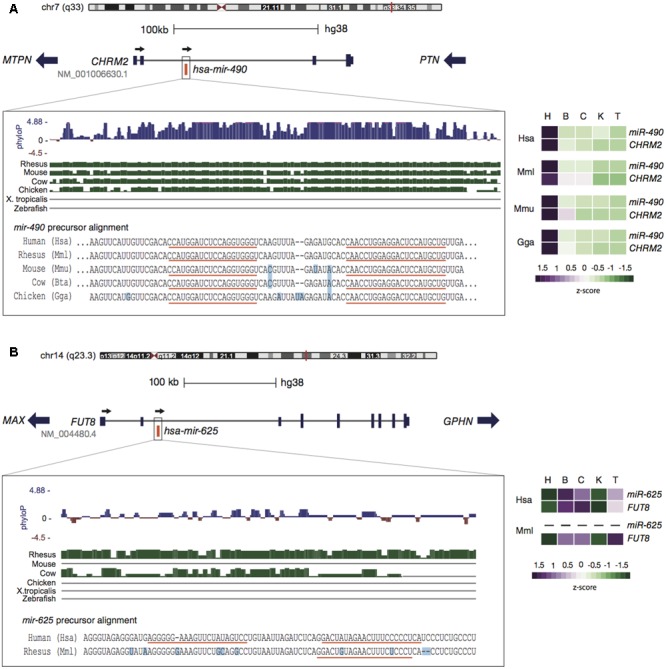
**Genomic context conservation of intragenic miRNAs. (A)** The human *miR-490* embedded within *CHRM2* reveals a highly conserved pattern in terms of sequence (left panel) and expression (right panel). Alignments from the UCSC genome browser indicate the preservation of *miR-490* throughout amniotes (green bars) with few differing bases (light blue squares) and identical mature sequences (orange lines). High-phyloP base scores indicate strong purifying selection on this region. *MiR-490* and *CHRM2* are co-expressed with highest levels in heart, a pattern conserved in other species. **(B)** The human *miR-625*, encoded within *FUT8*, has homologous sequences only in primates. The expression of *miR-625* follows its host pattern, with higher levels in brain and cerebellum, possibly reflecting rapid evolution. Expression of *miR-625* in rhesus was not detected. Expression data were obtained from Brawand et al. (2011) and Meunier et al. (2013) and processed in França et al. (2016). Tissues are: heart (H), brain (B), cerebellum (C), kidney (K), and testis (T).

Such kind of expression constraint linked to a conserved genomic context is clearly observed for *miR-490* and its host gene *CHRM2* (França et al., 2016). Homologous sequences of *miR-490* are found across amniotes, with identical mature sequences from human to chicken. Gene order and location of *miR-490* in the second intron of *CHRM2* are also preserved (**Figure 1A**). Although *miR-490* is annotated as intergenic in chicken, predicted transcripts with an intron overlapping *miR-490* are annotated. Expression analyses reveal a strongly conserved pattern among human, rhesus macaque, mouse, and chicken; indicating concomitant expression of *miR-490* and *CHRM2* (Shen et al., 2015) with highest abundance in heart (**Figure 1A**). The host gene is a muscarinic cholinergic receptor involved in acetylcholine-mediated cardiac chronotropic (heart rate) and inotropic (strength of muscle contraction) effects (Brodde and Michel, 1999), and it has been associated with cardiomyopathy (Zhang et al., 2008). Notably, dysregulation of *miR-490* is also reported in cardiac disease (Cooley et al., 2012) and is involved with proliferation of human coronary artery smooth cells (Sun et al., 2013), suggesting an important functional connection between *miR-490* and *CHRM2*.

As mentioned earlier, the transcriptional environment of host genes may act as a key factor to promote the expression of newly emerged miRNAs. This phenomenon is well illustrated by the primate-specific *miR-625* encoded within *FUT8* (**Figure 1B**). This host gene is a fucosyltransferase well-conserved throughout animals (Costache et al., 1997; Juliant et al., 2014) that catalyzes fucosylation of glycoproteins, which is essential for activating growth factor receptors (Liu et al., 2011), while its deletion has lethal effects in mice (Wang et al., 2005). *FUT8* is ubiquitously expressed in human tissues (Mele et al., 2015) and *miR-625* seems to follow its host expression pattern (**Figure 1B**). Considering the young evolutionary age of *miR-625*, its expression levels and breadth are unusually high, thus being frequently altered in different types of cancer (Zhou et al., 2014; Zheng et al., 2015). It is interesting that *miR-625* has emerged as a promising predictive biomarker in colorectal cancer (Verma et al., 2015; Rasmussen et al., 2016), exhibiting strong association with oxaliplatin (a chemotherapeutic agent used in the treatment of metastatic colorectal cancer) resistance (Rasmussen et al., 2016).

Another singular feature of miRNAs is their frequent occurrence in clusters, originated through tandem or non-local duplications or by *de novo* mutations either in introns or intergenic regions (Berezikov, 2011). Such genomic organization is prone to greatly affect the evolution of newly emerged miRNAs. According to Wang et al. (2016), members of the same cluster tend to exhibit coordinated expression and to target overlapping sets of genes. The authors proposed that clustering arrangement and by developing functions related to the pre-existing miRNAs in the same cluster would help the initial survival of these young miRNAs, until the cluster is settled up by purifying selection. Otherwise, the most usual fate of *de novo* newly emerged miRNAs would to undergo rapid degeneration. In further support of this “functional co-adaptation” model, clustered young miRNAs indeed present significant signs of adaptive changes that probably drive them to functional constraints associated with the older members of the cluster (Wang et al., 2016).

## miRNA–Target Interactions: Functional and Evolutionary Implications

**FIGURE 2:**
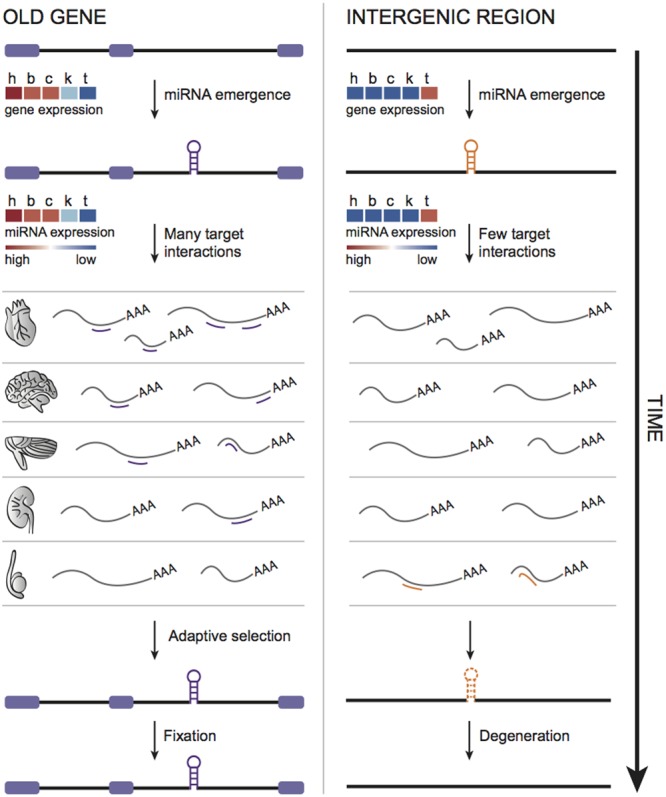
**Model of miRNA evolution.** Young miRNAs emerged within old genes are expressed in more tissues and, therefore, could interact with diverse set of targets, possibly enhancing the chances of functionalization and fixation through time. In contrast, as young intergenic miRNAs tend to be tissue-specific (likely expressed in testis), very limited target interactions could contribute to their faster degeneration.

If a recently emerged miRNA is expressed and integrated into regulatory networks through consistent and biologically relevant target interactions, it will have more chances to become functional and be retained afterward over long periods (Chen and Rajewsky, 2007; Lyu et al., 2014). Therefore, young miRNAs originated in a genomic context able to boost their expression in multiple tissues would favor target recognition. This idea is consistent with the previous observation that young miRNAs emerged within old host genes are expressed in more tissues and tend to have more predicted targets compared to young intergenic ones (França et al., 2016). We, therefore, suggested a miRNA evolution model that takes into account not only the miRNAs themselves, but also their genomic context (França et al., 2016) (**Figure 2**). Hence, young miRNAs (or “proto” miRNAs) hosted by old genes would gain higher expression breadth benefited by their host’s transcriptional activity, thus enabling many target interactions that, at first glance, are mostly neutral (Chen and Rajewsky, 2007; Nozawa et al., 2016), but could be stabilized by natural selection over time. On the other hand, as young intergenic miRNAs tend to have narrower expression, and apparently less targets to interact with, they could undergo faster degeneration (**Figure 2**). This degeneration scenario is also most likely to happen with miRNAs emerged within young hosts, because of their general tissue-specific expression signature (França et al., 2016).

Evolutionary sequence conservation has been successfully introduced to reduce the number of false-positive and to increase the signal-to-noise ratio in target predictions. Instead of helping identifying conserved pathways and relationships among miRNAs and their targets (Hausser and Zavolan, 2014), this requirement comes with a drawback, since it can only be applied to miRNAs and target genes that have conservation data available and which are not species-specific. Indeed, a recent study demonstrated that target sites identified by cross-linking immunoprecipitation data are rarely conserved between distantly related species, but extensive conservation is observed between closely related ones (Xu et al., 2013). Even when considering species-specific sites, there is evidence of selective constraints compared to non-target sites across the 3′UTR region, suggesting that most of non-conserved targets might be functional at least for a short evolutionary period. A striking example of this condition is the human-specific target site for *miR-183* in the 3vUTR of the transcription factor *FOXO1*, whose regulation altered FOXO1-dependent phenotypes, such as proliferation and migration, in a species-specific manner (McLoughlin et al., 2014). Despite of the recent advances on the characterization of operating mechanisms that guide miRNA–target interactions, we are only on the verge of understanding how newly emerged miRNAs in different genomic contexts are integrated into regulatory networks, as well as how their novel target interactions contribute to phenotypic plasticity.

## Population Biology Perspective for the Genome Architecture of miRNAs

Population biology studies at the genome level have been proved to be promising tools, enhancing our understanding on how genetic elements are interconnected spatially and temporally (Barrón et al., 2014; Sudmant et al., 2015). Most of miRNA population studies have focused on the impact of single nucleotide variants localized inside the seed and the mature regions to analyze conservation patterns, target diversification, and differential disease susceptibility (e.g., Barbash et al., 2014; Rawlings-Goss et al., 2014; Gallego et al., 2016). Except for few studies of miRNA expression quantitative trait loci (e.g., Huan et al., 2015), the evolution of miRNA genomic architecture has not been deeply investigated using a population biology framework.

It is still unknown if variation in miRNAs sequence, expression, and target sites across populations are more relevant for uncovering the mechanisms of phenotypic evolution and disease than other genetic variation. On one hand, due to its folded structure and small size, miRNAs are more likely to emerge *de novo* than novel protein coding genes (Berezikov, 2011). Diversification of miRNA target repertoire may be more prone to appear as result of simple sequence modifications such as direct mutation, seed or hairpin shifting, and arm switching (Berezikov, 2011). Therefore, variation on miRNA-binding sites indeed can lead to phenotypic innovation, as exemplified by the lineage diversification of cichlid fishes (Loh et al., 2011; Franchini et al., 2016). On the other hand, as target mRNAs can be regulated subtly by several miRNAs, detecting phenotypical effects by population variation seems to be harder than for genetic variation in regulatory or coding regions. Indeed, most of single nucleotide polymorphisms (SNPs) involved in the creation of novel miRNA target sites does not correlate with phenotypic differences among humans (Saunders et al., 2007).

Nonetheless, it is possible that genomic comparisons of different individuals can give insights on the origination process of miRNAs, as previously done for other genetic elements (Hatcher, 2000; Schlötterer, 2015). For instance, the basis of retrogene origination in metazoans has been recently deciphered through *Drosophila* population data. Flanking regions signatures of polymorphic retrocopies revealed that long terminal repeat (LTR) retrotransposons have mediate their formation (Tan et al., 2016). miRNAs are mostly originated *de novo* or by duplication (Meunier et al., 2013), but mechanistic details on how those processes occur are still unknown. Population genomics might help uncover those components through the identification of mutational signatures attached to polymorphic miRNAs that are usually erased by time and throughout their fixation.

In addition, comparing fixed patterns present in different species to polymorphic states observed in a group of individuals are useful tools for contrasting genomic features driven by natural selection to patterns produced by mutation bias (Long et al., 2013). Notable, this type of comparison helped to support the hypothesis in which natural selection drives retrogene duplication from the X chromosome to the autosomes in *Drosophila* and humans (Schrider et al., 2011, 2013; Navarro and Galante, 2015). Therefore, the analyses of different human populations can give further support to the adapted pattern of miRNAs organized inside old protein coding host genes.

Furthermore, as miRNAs expression and targeting has been shown to be implicated in a wide of human diseases (Mendell and Olson, 2012), seed, and mature region variants found among ethnic populations become clinically important (Rawlings-Goss et al., 2014). More specifically, there are distinct miRNA profiles in diseases between African and European descendants (e.g., Huang et al., 2011; Heegaard et al., 2012) which could be responsible for differences among those populations in susceptibility to diseases, drug sensitiveness, and biomarker diagnostics (Rawlings-Goss et al., 2014). Therefore, should worth investigating if ethnic group variation on miRNA genomic context have also significant role in human health.

From the discussion above, it turns out that the genomic context, as an outcome of natural selection, imposes evolutionary constraints to maintain the structural and functional integrity of its genetic elements. Moreover, it can also propel the evolutionary fate of new elements that arise in a suitable environment, eventually accelerating the process of functionalization. Therefore, evolutionary models tackling the 3D chromatin organization will be of extreme value to pursue the general principles that afford those processes take place throughout genomes.

## Author Contributions

GSF and MDV conceived the study. GSF, LCH, PAFG, and MDV wrote the manuscript. All authors revised and approved it for publication.

## Conflict of Interest Statement

The authors declare that the research was conducted in the absence of any commercial or financial relationships that could be construed as a potential conflict of interest.
